# Treatment as prevention: preparing the way

**DOI:** 10.1186/1758-2652-14-S1-S6

**Published:** 2011-07-06

**Authors:** Brian Williams, Robin Wood, Victor Dukay, Wim Delva, David Ginsburg, John Hargrove, Martinus Stander, Robert Sheneberger, Julio Montaner, Alex Welte

**Affiliations:** 1 South African Centre for Epidemiological Modelling and Analysis (SACEMA), Stellenbosch, South Africa; 2 The Desmond Tutu HIV Centre, Institute for Infectious Disease and Molecular Medicine, Faculty of Health Sciences, University of Cape Town, South Africa; 3 Lundy Foundation, Denver, Colorado, United States of America; 4 International Centre for Reproductive Health, University of Ghent, Belgium; 5 Rural Development Consultant, KwaZuluNatal, South Africa; 6 Health Econometrix and Outcomes Research (heXor), Johannesburg, South Africa; 7 Institute for Human Virology, University of Maryland, Baltimore, USA; 8 British Columbia Centre for Excellence in HIV/AIDS, Providence Health Care, Vancouver, Canada

## Abstract

Potent antiretroviral therapy (ART) reduces mortality and morbidity in people living with HIV by reducing viral load and allowing their immune systems to recover. The reduction in viral load soon after starting ART has led to the hypothesis that early and widespread ART could prevent onward transmission and therefore eliminate the HIV epidemic in the long term. While several authors have argued that it is feasible to use HIV treatment as prevention (TasP), provided treatment is started sufficiently early, others have reasonably drawn attention to the many operational difficulties that will need to be overcome if the strategy is to succeed in reducing HIV transmission. Furthermore, international public health policy must be based on more than theoretical studies, no matter how appealing. Community randomized controlled trials provide the gold standard for testing the extent to which early treatment reduces incidence, but much still needs to be understood and the immediate need is for operational studies to explore the practical feasibility of this approach. Here, we examine some of the issues to be addressed, the obstacles to be overcome, and strategies that may be necessary if TasP is to be effective. Studies of this kind will provide valuable information for the design of large-scale trials, as well as essential information that will be needed if early treatment is to be incorporated into public health policy.

## Introduction

An estimated 22 million people are living with HIV in sub-Saharan Africa [1]. Some will die as a result of their infections, but those who survive will need to be maintained on treatment at an annual *per capita* cost of approximately US$1500, or a total of US$660 billion over 20 years, although costs are likely to decline in future [2,3]. Even if they are left to die untreated, they will still need healthcare before they die and the cost of treating their AIDS-related illnesses in sub-Saharan Africa may run to about US$5000 per person [4] or about US$110 billion in total. Furthermore, each year there are more than twice as many new infections as there are people starting antiretroviral therapy (ART) [5]. While many lives have been saved using ART, the current situation is not financially sustainable and ways must be found to bring the epidemic to an end.

For long-term control of the HIV epidemic, the pertinent question is this: how can we stop HIV transmission? Many viral diseases can be prevented by vaccination, but for the AIDS retrovirus, the prospect of an effective vaccine remains elusive [6-8]. Abstaining from sex gives complete protection, but behaviour change programmes have had limited success [9,10]. The evidence that treating sexually transmitted infections reduces HIV incidence remains equivocal [11]. While male circumcision has been shown to be efficacious and is now being made available in much of Africa [12], it reduces the risk of HIV-infection by about 60% and only in men [13]. Condoms are effective, if used consistently and correctly, and promoting condoms among sex workers has yielded positive results [14], but condom use remains limited [15]. Vaginal microbicides [16-18] and pre-exposure prophylaxis [19] both give about 50% protection to un-infected people, but are most useful only in those at particularly high risk.

For those who start ART early, are fully compliant and have access to the best possible drug regimens, these drugs can reduce a person’s viral load by five orders of magnitude [20,21], dramatically reducing their infectiousness to others [22-26], and keeping their viral loads at very low levels for years and possibly for decades [20,21]. Where it is available, antiretroviral treatment has reduced mortality rates, but has not significantly reduced community-level sexual transmission because ART is provided mainly in the late stages of infection [27].

Using HIV treatment as prevention (TasP), in combination with other methods of prevention that have been shown to work, including male circumcision, vaginal microbicides and pre-exposure prophylaxis [19,28,29], offers the possibility of rapidly reducing HIV transmission. In this paper, we explore some operational research issues that need to be addressed as a matter of urgency if we are to consider using treatment as the primary means of stopping transmission and potentially ending the epidemic with a focus on heterosexual transmission in generalized epidemics.

## Discussion

### Treatment as prevention

Evidence supporting TasP has been outlined in a number of papers [3,30-32]; the arguments against using it have generally been concerned with practical issues of implementation, including stigma, acceptability, compliance, side effects, viral load suppression, viral rebound, treatment failure and drug resistance [33-36]. To these, we may add the importance of ensuring that a reliable and sustainable drug supply chain is in place, that the tools for monitoring the impact of TasP on both individual patients and the population are available, and that the short- and long-term costs of using TasP can be met [3].

Public health policy should be based on proven strategies, and amassing a body of evidence to support a new strategy is the necessary first step. Mathematical models, based on such data, can then provide support for pursuing the idea and can be used to explore alternative strategies, identify potential obstacles and design trials [3,17,37,38]. Field trials can then be carried out to establish the feasibility, or proof of concept, under realistic conditions, and to demonstrate impact at an individual level [25]. If possible, and only if appropriate and ethically acceptable, the final step is to carry out community randomized controlled trials to demonstrate the population-level impact directly.

Randomized controlled trials are the gold standard for testing new health interventions. However, a trial of TasP would need community, not individual, randomization, with the outcome being the incidence of infection. Trials of this kind can and have been done, but they typically require about six or more pairs of well-matched communities [39,40]. With an assumed HIV incidence of, say, 1% to 2% per total population per year, they require about 10,000 or more people in each community to measure a reduction in incidence of about 50%. The logistics and costs of running such a trial are substantial, and a trial of this kind is likely to take at least five years from start to finish. If places can be found where baseline demographic and HIV trend data are available, and if the funding can be found, community randomized controlled trials should be encouraged. When that time comes, it will be necessary to ask if there is still equipoise concerning the potential benefits to individuals of early treatment as this will determine whether or not such a trial is ethical.

More immediately, and perhaps of more practical relevance, feasibility or proof-of-concept studies could be started almost immediately and would provide valuable insights and lessons concerning the efficacy of a control strategy based on TasP. The *sine qua non* for the long-term success of this strategy will be to create community ownership, and to ensure that people understand that HIV can become a manageable chronic disease, that with ART they can live normal, healthy lives while being much less likely to infect their partners, and that eventually, this could eliminate new HIV infections from their community.

The primary goal of feasibility or proof-of-concept studies would be to show that it is possible to stop new HIV infections, whether sexually or vertically transmitted, through regular HIV testing and immediate provision of ART in a defined community.

However, a number of operational challenges would need to be met, and key questions to be answered would include: 1) Will the approach be acceptable to communities where it is offered? Can it reduce stigma and discrimination? 2) Will people agree to be tested annually for HIV? How best can this be achieved? 3) Will HIV-positive people agree to start ART at a very early stage of infection? 4) Will there be significant side effects? 5) If so, will this reduce compliance? 6) Will treatment lead to good viral load suppression? 7) Does good viral load suppression eliminate transmission? 8) Will making ART very widely available increase or decrease the extent to which people engage in risky sexual behaviour? 9) Will there be increases in either acquired or transmitted drug resistance? 11) Will it be possible to maintain people on treatment in communities with frequent in and out migration? 12) Is it affordable and can the costs be significantly reduced by relying on community support and engagement? 13) Will it be possible to use a substantial public health intervention, such as this, to create jobs in poor communities? 14) Can the medical infrastructure be ramped up quickly to deal with all the new patients on ART? 15) Can social media be used to communicate the benefits of treatment?

Ideally, independent proof-of-concept studies to explore the use of TasP should be carried out in several communities with different geographical, social, demographic and epidemiological characteristics. In the worst-affected countries in southern and east Africa, up to 10% of the entire population may be infected with HIV. In a community of 50,000 people, about 5000 will be HIV positive and about 500 more people will be infected each year. Testing 50,000 people a year is feasible (Submitted; Granich R *et al*: Achieving Universal Access for HIV and TB: potential prevention impact of an integrated multi-disease prevention campaign in Kenya), but maintaining 5000 people on ART at a cost of about US$1000 per person per year would cost US$5 million per year. If one considers a study lasting for two to three years and if one includes the cost of managing the study, as well as of the necessary monitoring and evaluation, the total cost would be of the order of US$20 million or more.

Since the success of TasP depends on achieving high rates of coverage and low rates of drop out, the target might be to ensure that fewer than about 5% drop out each year. With 5000 people on ART, a 5% rate can be measured with a precision of about 0.6%. Assuming that mortality in the cohort is less than 1.0% per year, this could be measured with a precision of 0.3%. If half the HIV-positive people have a regular partner and about half of infected couples are discordant, there will be about 800 discordant couples. In discordant couples not on ART, the transmission rate is typically about 5% per year [22-24], so that one would expect between 40 and 80 infections per year. If, as anticipated, ART reduces transmission by 95%, the number of transmissions in discordant couple should fall to between 2% and 4% per year, a difference that could be measured.

### Operational challenges

The four key strategies underlying such a trial would be: community empowerment, HIV testing, ART provision, and monitoring and evaluation. Each strategy involves important operational challenges that would need to be met.

#### Community empowerment

Unless the people living in the affected communities have a sense of ownership of the project, it is unlikely to succeed and to be sustained. To help to achieve this, “gate-keepers” in the community would have to be identified, community forums would have to be established, and representatives of young people, school teachers and principals, and religious and political leaders would have to be engaged. As far as possible, community members should be used to support clinical care, treatment, monitoring and evaluation, and to tackle stigma and discrimination [41].

Monitoring systems would be needed to evaluate people’s understanding of HIV. Indicators would be needed to measure acceptance of HIV testing, enrolment on ART, communication among couples about HIV status, percentage of couples tested, decreases in high-risk behaviour, increases in male circumcision, earlier engagement into HIV treatment services for HIV-positive people, especially pregnant women and tuberculosis patients, and male involvement.

Individual incentives for members of the community performing tasks integral to the ultimate success of TasP should be provided, but incentives should be tiered and based on performance goals measured through the community monitoring systems and indicators.

#### HIV testing

Since the beginning of 2004, HIV tests have been offered as a routine part of checkups in public and private clinics in Botswana [42] and could be complemented with regular testing campaigns. Data collected in the testing process would be used to determine the HIV incidence and to monitor changes over time. All testing should result in referral to appropriate HIV services, such as male circumcision, HIV prevention, risk reduction, family planning, and ART for HIV-positive people. Ideally, all those at risk of HIV in the community, including children exposed to mother to child trans mission, should be tested within the first year. HIV-negative people may be scheduled for retesting after three months and then annually. During the first year, pooled plasma RNA sampling of HIV negative cases can be used to provide estimates of acute infection rates in the community, providing the opportunity to determine whether this is a significant driver of new infections requiring particular attention.

#### ART provision

HIV-positive patients will have to be referred for immediate counselling, evaluation and initiation on ART, regardless of CD4 count or clinical staging. If financial and human resources are limited, one could consider giving priority to those with high viral loads as they have the shortest survival [43,44] and are the most infectious to others [22-24]. Patients on ART must be monitored for toxicity and side effects of ART. Routine samples will be collected to evaluate HIV resistance, viral load and CD4^+^ cell counts. Viral load monitoring is critical for testing the impact of TasP and should be carried out six weeks after ART initiation and then every six months for people on ART.

Expanded training for healthcare workers will be needed. An increase in the workforce for ART provision will be critical as expanded numbers of people come forward for testing and referral services. This workforce should be drawn from the pool of current healthcare providers, augmented by community workers trained to provide treatment support and to monitor and evaluate aspects of the project. Stable patients on ART, requiring lower levels of care and monitoring, could be managed through alternative healthcare sites but with increased community workforce support. Laboratory support would have to be in place. The community workforce will be essential for tracking patients on ART, ensuring that adherence with ART is documented, reducing the number of defaulters, and monitoring migration in and out of the community.

#### Monitoring and evaluation

When scaling up testing and early treatment, data management will be essential. Biometric fingerprinting could be used to prevent duplication of testing and to monitor patients. An integrated data system should be used to track patients on ART, monitor testing rates, HIV status, referrals for ART, pharmacy pick ups, laboratory results, and follow-up care. To preserve confidentiality, electronic ID cards may be used to reduce the risk of increased stigmatization; when the card is read electronically, the person’s status will be known only to the healthcare providers. The data should be used not only to answer the key research questions, but also to provide feedback to the community.

The most important outcomes to be measured will be acceptance of testing, agreement to start treatment, compliance, side effects, viral load suppression, viral rebound, drug resistance, residual transmission between discordant couples, risk compensation, and the impact on opportunistic infections and mortality.

### Planned and ongoing studies

Interest in using TasP has grown rapidly in the past two years [45] and a number of studies are in various stages of planning and development. Some involve viral load testing which, although not widely available in most developing countries, could be used to better target those who are at greatest risk of dying and are most likely to transmit the virus.

The Mochudi project in Botswana [46] will combine community-wide HIV prevention, including male circumcision, with a “test-and-treat” strategy. HIV-positive people with CD4^+^ cell counts above 250 cells/mm^3^ will be offered three-drug ART if their viral loads are greater than 50,000 RNA copies/mL, while those with CD4^+^ cell counts below 250 cells/mm^3^ will be referred for ART through the public programme. HIV incidence and molecular methods to elucidate transmission pathways will be used to evaluate the impact of the trial, which will last for five years.

In the iTLC (International Testing and Linkage to Care) study, the HIV Prevention Trials Network and Family Health International are planning a three-arm, multi-site community randomized trial [46] comparing: a) the local standard of care; b) enhanced HIV testing and linkage to care with ART initiation based on the local standard of care; and c) enhanced testing and linkage to care with ART provided to all HIV-infected patients who have plasma HIV-1 RNA greater than 50,000 copies/mL.

The TLC (Test and Link to Care) study, also coordinated by the HIV Prevention Trials Network, will determine the feasibility of a community-based, enhanced test and link-to-care strategy in Washington DC and The Bronx, New York [47]. This study will focus on expanded HIV testing, linkage to care, viral load suppression, prevention for positives, and patient and provider surveys.

The PopART (Population Effects of Anti-Retroviral Therapy) trial is being developed by scientists based in the United Kingdom and in Africa to see if the widespread use of ART for all adults testing HIV positive could substantially reduce HIV transmission [48].

TTEA (Test and Treat to End AIDS) is a feasibility study of annual testing and immediate treatment in the Western Cape Province of South Africa, being planned by scientists in South Africa, the United States and Canada [46,49].

ANRS (Agence Nationale de Recherches sur le SIDA) 12249-TasP is planning a pilot study of TasP which, if successful, will be developed into a community randomized controlled trial. It will be done by scientists from the University of Bordeaux, the Hôpitaux Universitaires de Genève, and the Africa Centre for Health and Population Studies in South Africa [50].

Stop AIDS Now!, a Dutch non-governmental organization, with support from the South African Centre for Epidemiological Modelling and Analysis (SACEMA) and the Clinton Health Access Initiative (CHAI), has been granted money from the Dutch Lottery Fund to start a project in Swaziland to: ensure that, within three years, there is universal access to treatment under current UNAIDS/WHO guidelines; evaluate the impact of achieving this; and carry out a proof-of-concept study for treatment-centred prevention [51].

The SEARCH collaboration is performing pilot studies in preparation for a community randomized study to evaluate health, economic and education outcomes of universal ART (test and treat at all CD4 cell counts) in three east African countries: Uganda, Kenya and Tanzania (Diane Havlir, personal communication).

The NIH (National Institutes of Health, USA) has awarded grants to 12 research teams as part of a five-year effort to study HIV prevention, testing and treatment for individuals in jails and prisons. The programme aims to link inmates to care – and, if appropriate, antiretroviral therapy – while incarcerated, with continued follow up and support after they are released and return to their communities [52].

## Conclusions

TasP offers the chance of significantly reducing HIV transmission and eventually bringing the HIV epidemic to an end. The World Health Organization previously recommended starting ART for all people with CD4^+^ cell counts below 200 cells/mm^3^[53], but, in 2010, this was raised to 350 cells/mm^3^[5]. The United States Department of Health and Human Services and the International AIDS Society-USA guidelines now recommend consideration of ART even when the CD4^+^ cell count is greater than 500/mm^3^[54], while in San Francisco the public health system recommends that all HIV-positive people should be offered immediate access to ART [56]. Modelling studies show that each of these increases, 200 cells/mm^3^ to 350 cells/mm^3^ to 500 cells/mm^3^ to immediate treatment, will further reduce the incidence of HIV, as well as of AIDS-related tuberculosis [17].

A number of studies to investigate the impact of TasP are being planned or have started, and these will provide important and valuable information for the design of more rigorous trials. They will also inform public health policy regarding the management of HIV, regardless of whether or not they show that TasP could stop transmission. However, an ambitious programme of TasP would, in its initial phases, require significant increases in funding, and it will be important to investigate the financial demands and economic impact of TasP. If ways can be found to ensure that those carrying out these projects remain in close contact and exchange ideas, plans and results, the work will proceed more rapidly and efficiently.

In parallel with feasibility studies, it will be important to develop cost-benefit analyses to compare the current HIV/AIDS strategy with one involving much earlier treatment, and, indeed, to examine the impact of targeting TasP at those in greatest need and at those who, for behavioural or biological reasons, are most likely to infect others. Costs will include direct healthcare investments needed to implement the strategy, including the cost of drugs, infrastructure, logistics and human resources. Benefits will include the reduction in new infections, including opportunistic infections, and deaths, reduced demands placed on the healthcare system, and the benefits to society of keeping young adults alive.

The present situation, in which increasing numbers of HIV-positive people need to be maintained on expensive drugs, which must be taken regularly and for life, is not sustainable. Ways must be found to significantly reduce, and perhaps even eliminate, transmission. Where possible, TasP should be backed up and supported by other methods of prevention, including the promotion of behaviour change, making condoms available and accessible, providing male circumcision services, promoting needle exchange programmes, couples counselling, addressing the needs of pregnant women, providing ART to people suffering from other opportunistic infections, especially tuberculosis, and providing pre-exposure prophylaxis to those at very high risk. However, much still needs to be learned and there will be many obstacles to overcome. But if the work starts now, we might hope to see significant and rapid reductions in transmission within the next decade.

## Competing interests

The authors declare that they have no competing interests.

## Authors’ contributions

All authors have contributed substantially to the development of the ideas discussed in this paper, have contributed to drafting and writing the manuscript, and have approved the final version.

## References

[B1] UNAIDS UNAIDS Report on the Global AIDS Epidemic 2010 http://www.unaids.org/documents/20101123_GlobalReport_em.pdf

[B2] MSF Campaign for Access to Essential Medicines Untangling the Web of Antiretroviral Price Reductions http://utw.msfaccess.org/background/aids_progress_under_siege

[B3] Granich RM Gilks CF Dye C De Cock KM Williams BG Universal voluntary HIV testing with immediate antiretroviral therapy as a strategy for elimination of HIV transmission: a mathematical model Lancet 2009 14 48 57 10.1016/S0140-6736(08)61697-9 19038438

[B4] Badri M Maartens G Mandalia S Bekker LG Penrod JR Platt RW Wood R Beck EJ Cost-efectiveness of highly active antiretroviral therapy in South Africa PLoS Med 2006 14 e4 10.1371/journal.pmed.0030004 16318413 PMC1298940

[B5] WHO Towards universal access: scaling up priority HIV/AIDS interventions in the health sector Progress report 2009 http://www.who.int/hiv/pub/2009progressreport/en/

[B6] McElrath MJ Immune responses to HIV vaccines and potential impact on control of acute HIV-1 infection J Infect Dis 2010 14 Suppl 2 S323 326 20846040 10.1086/655658 PMC2946178

[B7] Berkley S Bertram K Delfraissy J-F Draghia-Akli R Fauci A Hallenbeck C Kagame J Kim P Mafubelu D Makgoba MW Piot P Walport M Warren M Yamada T Esparza J Hankins C Johnston MI Lévy Y Manuel Romaris M Ahmed R Bernstein A The 2010 scientific strategic plan of the Global HIV Vaccine Enterprise Nat Med 2010 14 981 989 10.1038/nm0910-981 20823883

[B8] Barouch DH Korber B HIV-1 vaccine development after STEP Annu Rev Med 2010 14 153 167 10.1146/annurev.med.042508.093728 20059334 PMC2819364

[B9] Ross DA Changalucha J Obasi AI Todd J Plummer ML Cleophas-Mazige B Anemona A Everett D Weiss HA Mabey DC Grosskurth H Hayes RJ Biological and behavioural impact of an adolescent sexual health intervention in Tanzania: a community-randomized trial AIDS 2007 14 1943 1955 10.1097/QAD.0b013e3282ed3cf5 17721102

[B10] McCoy SI Kangwende RA Padian NS Behavior change interventions to prevent HIV infection among women living in low and middle income countries: a systematic review AIDS Behav 2010 14 469 482 10.1007/s10461-009-9644-9 19949847

[B11] White RG Orroth KK Glynn JR Freeman EE Bakker R Habbema JD Terris-Prestholt F Kumaranayake L Buve A Hayes RJ Treating curable sexually transmitted infections to prevent HIV in Africa: still an effective control strategy? J Acquir Immune Defic Syndr 2008 14 346 353 10.1097/QAI.0b013e318160d56a 18176323 PMC3776949

[B12] Lissouba P Taljaard D Rech D Doyle S Shabangu D Nhlapo C Otchere-Darko J Mashigo T Matson C Lewis D Billy S Auvert B A model for the roll-out of comprehensive adult male circumcision services in African low-income settings of high HIV incidence: the ANRS 12126 Bophelo Pele Project PLoS Med 2010 14 e1000309 10.1371/journal.pmed.1000309 20652013 PMC2907271

[B13] Auvert B Taljaard D Lagarde E Sobngwi-Tambekou J Sitta R Puren A Randomized, controlled intervention trial of male circumcision for reduction of HIV infection risk: The ANRS 1265 trial PLoS Med 2005 14 e298 10.1371/journal.pmed.0020298 16231970 PMC1262556

[B14] Park LS Siraprapasiri T Peerapatanapokin W Manne J Niccolai L Kunanusont C HIV transmission rates in Thailand: evidence of HIV prevention and transmission decline J Acquir Immune Defic Syndr 2010 14 430 436 20418773 10.1097/QAI.0b013e3181dc5dad

[B15] Foss AM Watts CH Vickerman P Heise L Condoms and prevention of HIV Br Med J 2004 14 185 186 10.1136/bmj.329.7459.185 15271806 PMC487722

[B16] Abdool Karim SS Results of effectiveness trials of PRO 2000 gel: lessons for future microbicide trials Future Microbiology 2010 14 527 529 10.2217/fmb.10.29 20353292

[B17] Williams BG Granich R De Cock K Glaziou P Sharma A Dye C Anti-retroviral therapy for the control of HIV-associated tuberculosis: modelling the potential effects in nine African countries Proc Natl Acad Sci USA 2010 14 17853 17854

[B18] Williams BG Abdool Karim S Gouws E Abdool Karim Q The epidemiological impact of Tenofovir gel on HIV in South Africa XVIII International AIDS Conference; July 18-23 2010; Vienna, Austria

[B19] Grant RM Lama JR Anderson PL McMahan V Liu AY Vargas L Goicochea P Casapia M Guanira-Carranza JV Ramirez-Cardich ME Montoya-Herrera O Fernandez T Veloso VG Buchbinder SP Chariyalertsak S Schechter M Bekker L-G Mayer KH Kallas EG Amico KR Mulligan K Bushman LR Hance RJ Ganoza C Defechereux P Postle B Wang F McConnell JJ Zheng J-H Lee J Rooney JF Jaffe HS Martinez Al Burns DN Glidden DV Preexposure chemoprophylaxis for hiv prevention in men who have sex with men N Engl J Med 2010 14 2587 2599 10.1056/NEJMoa1011205 21091279 PMC3079639

[B20] Simon V Ho DD HIV-1 dynamics in vivo: implications for therapy Nat Rev Microbiol 2003 14 181 190 10.1038/nrmicro772 15035022

[B21] Palmer S Maldarelli F Wiegand A Bernstein B Hanna GJ Brun SC Kempf DJ Mellors JW Coffin JM King MS Low-level viremia persists for at least 7 years in patients on suppressive antiretroviral therapy Proc Natl Acad Sci USA 2008 14 3879 3884 10.1073/pnas.0800050105 18332425 PMC2268833

[B22] Attia S Egger M Muller M Zwahlen M Low N Sexual transmission of HIV according to viral load and antiretroviral therapy: systematic review and meta-analysis AIDS 2009 14 1397 1404 10.1097/QAD.0b013e32832b7dca 19381076

[B23] Donnell D Baeten JM Kiarie J Thomas KK Stevens W Cohen CR Mclntyre J Lingappa JR Celum C Heterosexual HIV-1 transmission after initiation of antiretroviral therapy: a prospective cohort analysis Lancet 2010 14 2092 2098 10.1016/S0140-6736(10)60705-2 20537376 PMC2922041

[B24] Lingappa JR Hughes JP Wang RS Baeten JM Celum C Gray GE Stevens WS Donnell D Campbell MS Farquhar C Essex M Mullins Jl Coombs RW Rees H Corey L Wald A Estimating the impact of plasma HIV-1 RNA reductions on heterosexual HIV-1 transmission risk PLoS ONE 2010 14 e12598 10.1371/journal.pone.0012598 20856886 PMC2938354

[B25] Williams BG Lima V Gouws E Modelling the impact of ART on the epidemic of HIV Curr HIV Res 2011 in press 10.2174/157016211798038533 PMC3529404 21999772

[B26] del Romero J Marincovich B Castilla J Garcia S Campo J Hernando V Rodriguez C Evaluating the risk of HIV transmission through unprotected orogenital sex AIDS 2002 14 1296 1297 10.1097/00002030-200206140-00017 12045500

[B27] Keiser O Orrell C Egger M Wood R Brinkhof MW Furrer H van Cutsem G Ledergerber B Boulle A Public-health and individual approaches to antiretroviral therapy: township South Africa and Switzerland compared PLoS Med 2008 14 e148 10.1371/journal.pmed.0050148 18613745 PMC2443185

[B28] Padian NS Buve A Balkus J Serwadda D Cates W Jr. Biomedical interventions to prevent HIV infection: evidence, challenges, and way forward Lancet 2008 14 585 599 10.1016/S0140-6736(08)60885-5 18687456

[B29] Kurth AE Celum C Baeten JM Vermund SH Wasserheit JN Combination HIV Prevention: Significance, Challenges, and Opportunities Current HIV/AIDS Reports 2011 14 62 72 10.1007/s11904-010-0063-3 20941553 PMC3036787

[B30] Montaner JS Hogg R Wood E Kerr T Tyndall M Levy AR Harrigan PR The case for expanding access to highly active antiretroviral therapy to curb the growth of the HIV epidemic Lancet 2006 14 531 536 10.1016/S0140-6736(06)69162-9 16890841

[B31] Granich R Crowley S Vitoria M Lo YR Souteyrand Y Dye C Gilks C Guerma T De Cock KM Williams B Highly active antiretroviral treatment for the prevention of HIV transmission Journal of the International AIDS Society 2010 14 1 10.1186/1758-2652-13-1 20205768 PMC2822750

[B32] Granich R Crowley S Vitoria M Smyth C Kahn JG Bennett R Lo YR Souteyrand Y Williams B Highly active antiretroviral treatment as prevention of HIV transmission: review of scientific evidence and update Current Opinion in HIV AIDS 2010 14 298 304 10.1097/COH.0b013e32833a6c32 PMC3501989 20543604

[B33] Walensky RP Paltiel AD Losina E Morris BL Scott CA Rhode ER Seage GR Freedberg KA Test and treat DC: forecasting the impact of a comprehensive HIV strategy in Washington DC Clin Infect Dis 2010 14 392 400 10.1086/655130 20617921 PMC2906630

[B34] Wagner BG Kahn JS Blower S Should we try to eliminate HIV epidemics by using a 'Test and Treat' strategy? AIDS 2010 14 775 776 10.1097/QAD.0b013e3283366782 20177359 PMC2895980

[B35] Smith RJ Okano JT Kahn JS Bodine EN Blower S Evolutionary dynamics of complex networks of HIV drug-resistant strains: the case of San Francisco Science 2010 14 697 701 10.1126/science.1180556 20075214

[B36] Garnett GR Baggaley RF Treating our way out of the HIV pandemic: could we, would we, should we? Lancet 2009 14 9 11 10.1016/S0140-6736(08)61698-0 19038439

[B37] Dodd PJ Garnett GR Hallett TB Examining the promise of HIV elimination by 'test and treat' in hyperendemic settings AIDS 2010 14 729 735 10.1097/QAD.0b013e32833433fe 20154580 PMC2852517

[B38] Bacaer N Pretorius C Auvert B An age-structured model for the potential impact of generalized access to antiretrovirals on the South African HIV epidemic Bull Math Biol 2010 14 2180 2198 10.1007/s11538-010-9535-2 20349152

[B39] Hayes R Mosha F Nicoll A Grosskurth H Newell J Todd J Killewo J Rugemalila J Mabey D A community trial of the impact of improved sexually transmitted disease treatment on the HIV epidemic in rural Tanzania: 1. Design AIDS 1995 14 919 926 10.1097/00002030-199508000-00014 7576328

[B40] Hayes R Kapiga S Padian N McCormack S Wasserheit J HIV prevention research: taking stock and the way forward AIDS 2010 14 SuppI 4 S81 92 21042056 10.1097/01.aids.0000390710.04255.2b PMC3154649

[B41] Chang LW Alamo S Guma S Christopher J Suntoke T Omasete R Montis JP Quinn TC Juncker M Reynolds SJ Two-year virologic outcomes of an alternative AIDS care model: evaluation of a peer health worker and nurse-staffed community-based program in Uganda J Acquir Immune Defic Syndr 2009 14 276 282 10.1097/QAI.0b013e3181988375 19194316 PMC2662625

[B42] AVERT HIV & AIDS in Botswana http://www.avert.org/aids-botswana.htm

[B43] Williams BG Korenromp EL Gouws E Schmid GP Auvert B Dye C HIV infection, antiretroviral therapy, and CD4+ cell count distributions in African populations J Infect Dis 2006 14 1450 1458 10.1086/508206 17054076

[B44] Mellors JW Rinaldo CR Jr. Gupta P White RM Todd JA Kingsley LA Prognosis in HIV-1 infection predicted by the quantity of virus in plasma Science 1996 14 1167 1170 10.1126/science.272.5265.1167 8638160

[B45] WHO Consultation on Antiretroviral Treatment for Prevention of HIV Transmission Meeting Report. 2-4 November 2009; Geneva 2010

[B46] Lockman S Test-and-Treat Strategy for HIV in Resource-Limited Settings Medscape 2010 http://www.medscape.com/viewarticle/727536

[B47] TLC-Plus A Study to Evaluate the Feasibility of an Enhanced Test, Link to Care, Plus Treat Approach for HIV Prevention in the United States http://www.hptn.org/webdocuments/HPTN065/HPTN065ProtocolFINALVer1_01Mar10.pdf 10.1177/1740774517711682 PMC5639958 28627929

[B48] Department of Medicine PopART- Population effects of Anti Retroviral Therapy Imperial College, London http://www1.imperial.ac.uk/departmentofmedicine/divisions/infectiousdiseases/infectious_diseases/hiv_trials/popart/

[B49] Test and Treat to End AIDS Test-and-Treat Strategy to End AIDS http://www.ttea.info

[B50] ANRS Traiter toutes les personnes infectées par le VIH: un impact sur la pandémie? http://www.groupesida.ch/filrouge/archives/2010/11/anrs_traiter_toutes_les_person/

[B51] Stop AIDS Now! Ending New HIV Infections in Swaziland: A Catalytic Model for Southern Africa http://www.stopaidsnow.org/

[B52] NIH Unprecedented effort to seek, test, and treat inmates with HIV: NIH research to improve public health with focus on prison and jail systems across the United States 2010 http://www.nih.gov/news/health/sep2010/nida-23.htm

[B53] WHO Towards universal access: scaling up priority HIV/AIDS interventions in the health sector Progress report 2008 http://www.who.int/hiv/pub/2008progressreport/en/index.html

[B54] Gallant JE When to Start Therapy: Chronic HIV Infection Johns Hopkins HIV Guide http://www.hopkins-aids.edu/management/antiretroviral_therapy/when_to_start_therapy.html?contentlnstanceld=7666

[B55] Universal Antiretroviral Therapy Initiation: Guideline of the HIV/AIDS Division at San Francisco General Hospital Updated January 31 2010

